# A Hemi-*Hemi*-Hamate Osteochondral Graft: A Modified Hemi-Hamate Technique for a Unicondylar Defect

**DOI:** 10.1016/j.jhsg.2023.11.009

**Published:** 2023-12-27

**Authors:** Ignazio Marcoccio, Carolina Civitenga, Jacopo Maffeis, Andrea Minini, Michele Riccio, Pasquale Gravina

**Affiliations:** ∗ Hand Surgery and Microsurgical Peripheral Nerve Reconstruction Unit, Istituto Clinico Città di Brescia – Gruppo Ospedaliero San Donato, Via Bartolomeo Gualla, Brescia, Italy; † Department of General and Specialties Surgery, Department of Plastic and Reconstructive Surgery-Hand Surgery Unit, Azienda Ospedaliera Universitaria (University Hospital) “Ospedali Riuniti,” Ancona, Italy; ‡ Clinical Orthopedics, Department of Clinical and Molecular Science, Polytechnic University of Marche, Ancona, Italy

**Keywords:** Hand fracture, Hemi-hamate, Proximal interphalangeal joint

## Abstract

Articular comminuted fracture dislocations of the base of the middle phalanx represent a major challenge for the surgeon. The treatment goal is a nonpainful, stable, and functional proximal interphalangeal joint, which is achieved through concentric joint reduction and restoration of joint stability. Fracture pattern rarely results in sagittal bone loss involving the entire ulnar or radial pilon of the base of the second phalanx. In these cases, the choice of treatment can be particularly challenging as the loss of a pillar of the articular base causes angular deviation at the joint level, thus causing the loss of finger joint flexion and overlap of the adjacent finger. We present a novel nonvascularized osteochondral graft, which we named hemi-*hemi*-hamate osteochondral graft*,* a modified version of the traditional hemi-hamate arthroplasty, that is suitable for the reconstruction of bone loss involving the whole anteroposterior hemiarticular surface of the base of the P2.

The proximal interphalangeal joint (PIPJ) is a complex anatomical structure in which stability depends on the involved bone, ligaments, and volar plate. Articular comminuted fracture dislocations of the base of the middle phalanx represent a major challenge for the surgeon; however, despite prompt surgical treatment, the results are often unsatisfactory with outcomes including pain, PIPJ stiffness, and disability. The treatment goal is a nonpainful, stable, and functional PIPJ, which is achieved through concentric joint reduction and restoration of joint stability. Surgery is indicated in the cases of PIPJ dorsal fracture dislocation when the volar lip fragment involves more than 50% of the articular surface or in selected cases of 30% to 50% articular surface involvement.^1^ Different techniques have been described, from percutaneous pinning to free pedicle graft. In acute settings, it is of utmost importance to implement an optimized reconstructive treatment tailored according to the fracture pattern of the bony defect.^1^ Articular involvement is strictly predictive of joint instability.^2^ The comminuted fracture is most often confined to the volar lip of the base of the middle phalanx, producing a dorsal subluxation of the joint; nevertheless, a dorsal or central lip comminuted fracture is possible. Different treatment options have been described, such as open reduction internal fixation,^1^ volar plate arthroplasty, dynamic external fixation, and hemi-hamate arthroplasty.^3^ Fractures left untreated for more than 3 to 6 weeks are considered chronic; thus, the surgical approach for such cases is different and includes volar plate arthroplasty, silicon, or pyrocarbon arthroplasties and hemi-hamate techniques, which can be valuable for chronic fracture management. When the surgeon identifies a malunited fracture, salvage procedures such as joint replacement,^4^ hemi-hamate arthroplasty,^3^ or arthrodesis^5^ are the most widespread options. Free vascularized toe joint transfers yield the best results in range of movement recovery and are suitable for the reconstruction of wide defects throughout the whole articular surface despite requiring advanced microsurgical skills, causing severe donor site morbidity, and having a high rate of secondary procedures.^6^ A hemi-toe osteochondral graft and a lateral femoral condyle osteochondral graft^7^ are technically simpler solutions, despite both requiring a secondary limb as a donor site.

Fracture pattern rarely results in sagittal bone loss involving the entire ulnar or radial pilon of the base of the second phalanx. In these cases, the choice of treatment can be particularly challenging as the loss of a pillar of the articular base causes angular deviation at the joint level, thus causing the loss of finger joint flexion and overlap of the adjacent finger. We report a case of an untreated impaction fracture of the PIP joint that evolved into a sagittal bone defect of the entire anteroposterior ulnar base of the P2 of the fourth finger and was treated with a hemi-*hemi*-hamate osteochondral graft, a modified version of the traditional hemi-hamate arthroplasty.

## Case Report

**Figure 1:**
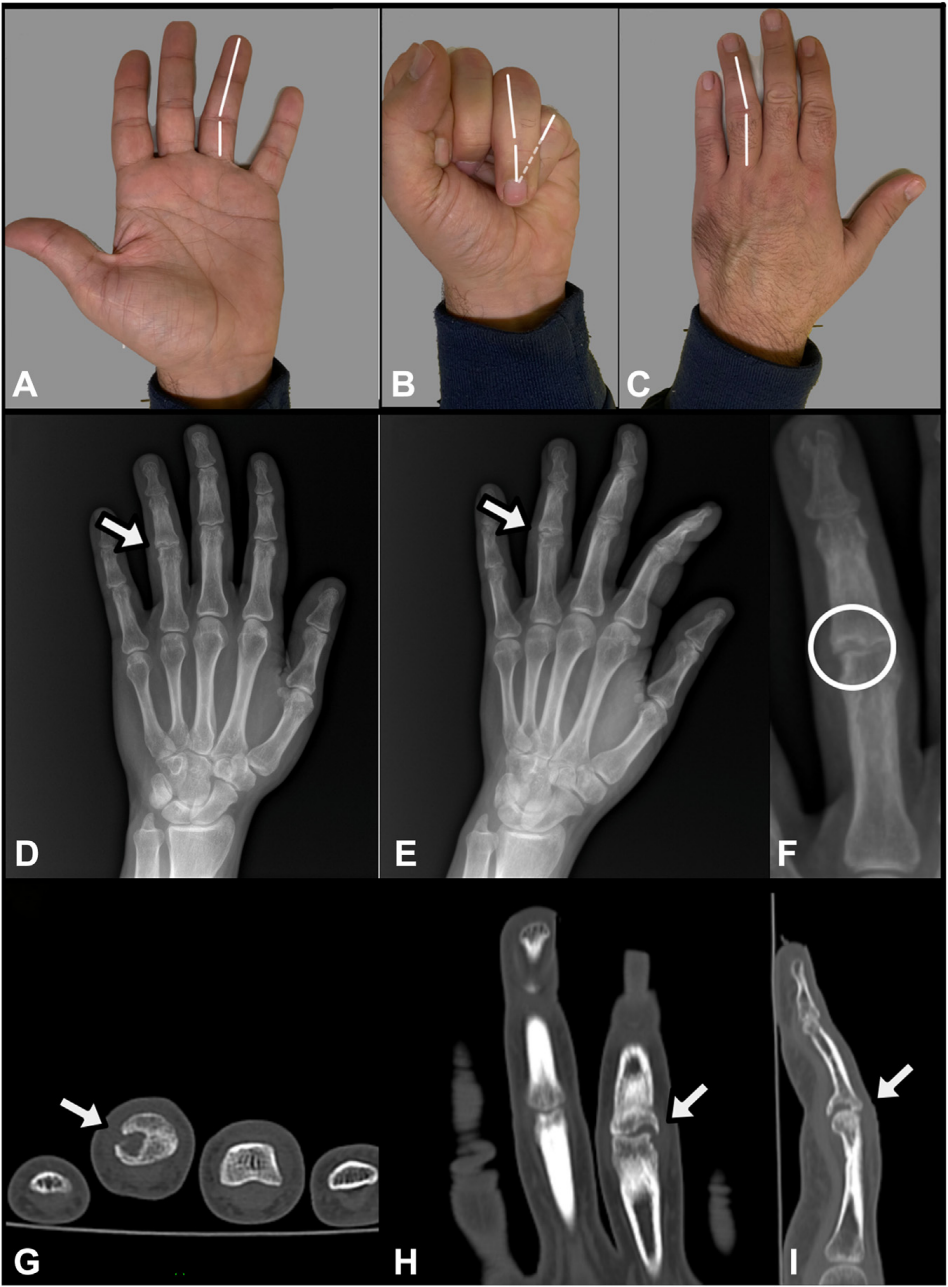
Preoperative assessment. **A** Palmar view of angular PIPJ ulnar deviation of 15° at full extended digits. **B** Palmar view of fourth finger overlapping fifth during flexion. **C** Dorsal view of fourth finger angular deviation. **D** Preoperative anteroposterior x-ray. We can notice PIPJ defect (arrow). **E** Preoperative oblique x-ray. **F** Detail of bone defect. **G** Preoperative CT scan axial view. **H** Preoperative CT scan transversal view. **I** Preoperative CT scan coronal view.

A 38-year-old hand worker presented with pain in the fourth finger of the left hand resulting in limited PIPJ flexion to the outpatient department at which the first author is employed. The patient reported that 2 months ago, the finger became hooked in the leash while taking the dog for a walk, thus causing dorsoulnar subluxation. Trauma severity was underestimated in patients who were not referred to first aid. When the patient first came to our clinic, the physical examination showed a fourth finger ulnar deviation of approximately 15°, limited and painful motion and overlap with the fifth finger during flexion (Fig. 1A–C). The patient underwent an x-ray, which showed a PIPJ fracture with loss of half of the ulnar base of the P2 (Fig. 1D–F). A CT scan confirmed complete bone resorption (Fig. 1G–I). Considering the patient and fracture characteristics, his job, and time since the trauma, we proposed a hemi-hamate autologous bone graft, which we renamed “the hemi-*hemi*-hamate technique.” First author performed the surgery.

**Figure 2:**
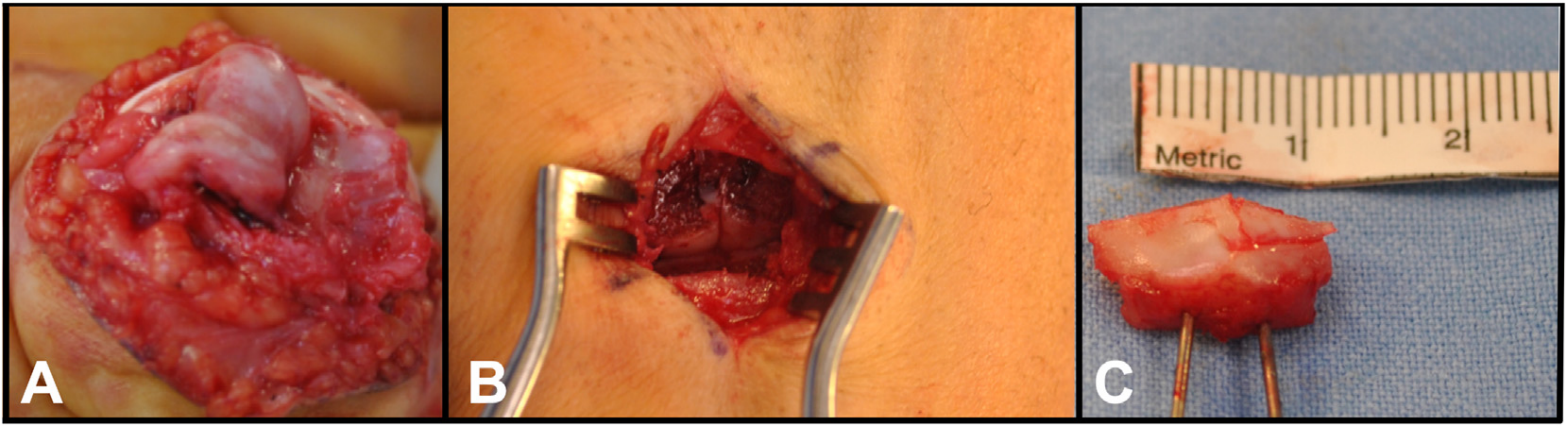
Surgical technique. **A** PIPJ exposure throw “shotgun approach”. **B** Graft harvesting through small part of fourth and fifth metacarpal base, deeper than usual harvest. **C** Graft harvested.

**Figure 3:**
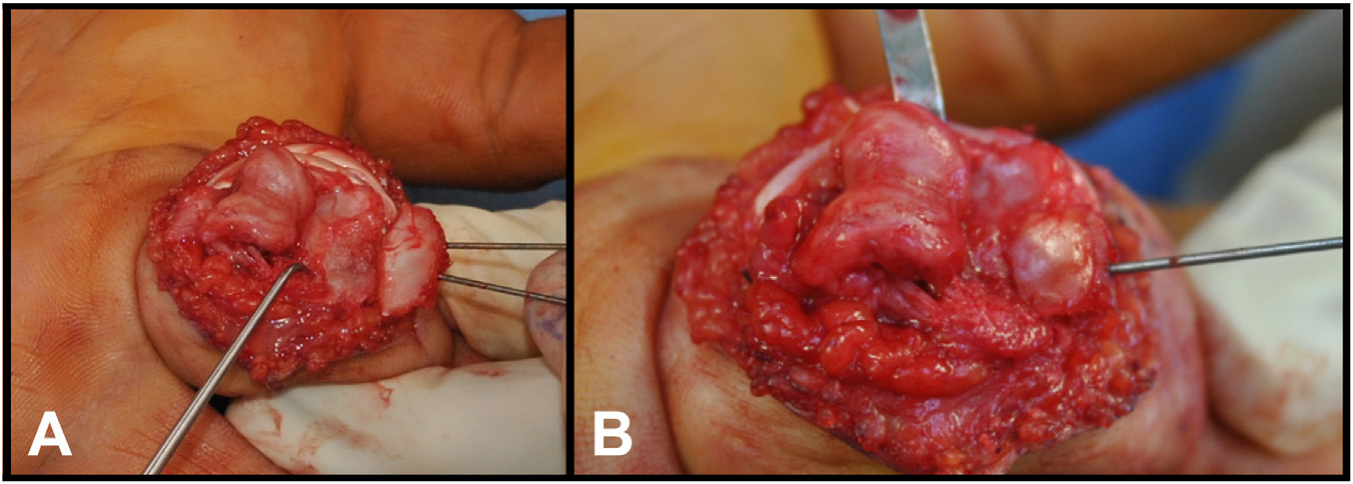
**A** The graft is oversized to facilitate the recontouring. **B** The graft is shaped to match the bone defect.

**Figure 4:**
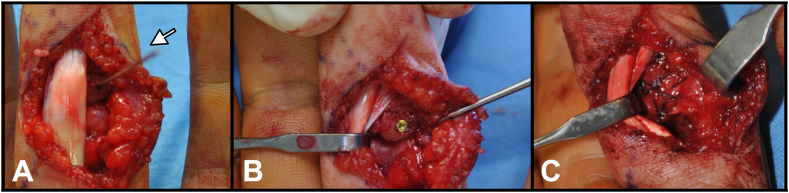
**A** The arrow shows the K-wire for reduction and for screw direction. **B** Fixation with a 1.2 mm compression screw. **C** A3 pulley sutured above the volar plate to reinforce its “buttress” effect.

**Figure 5:**
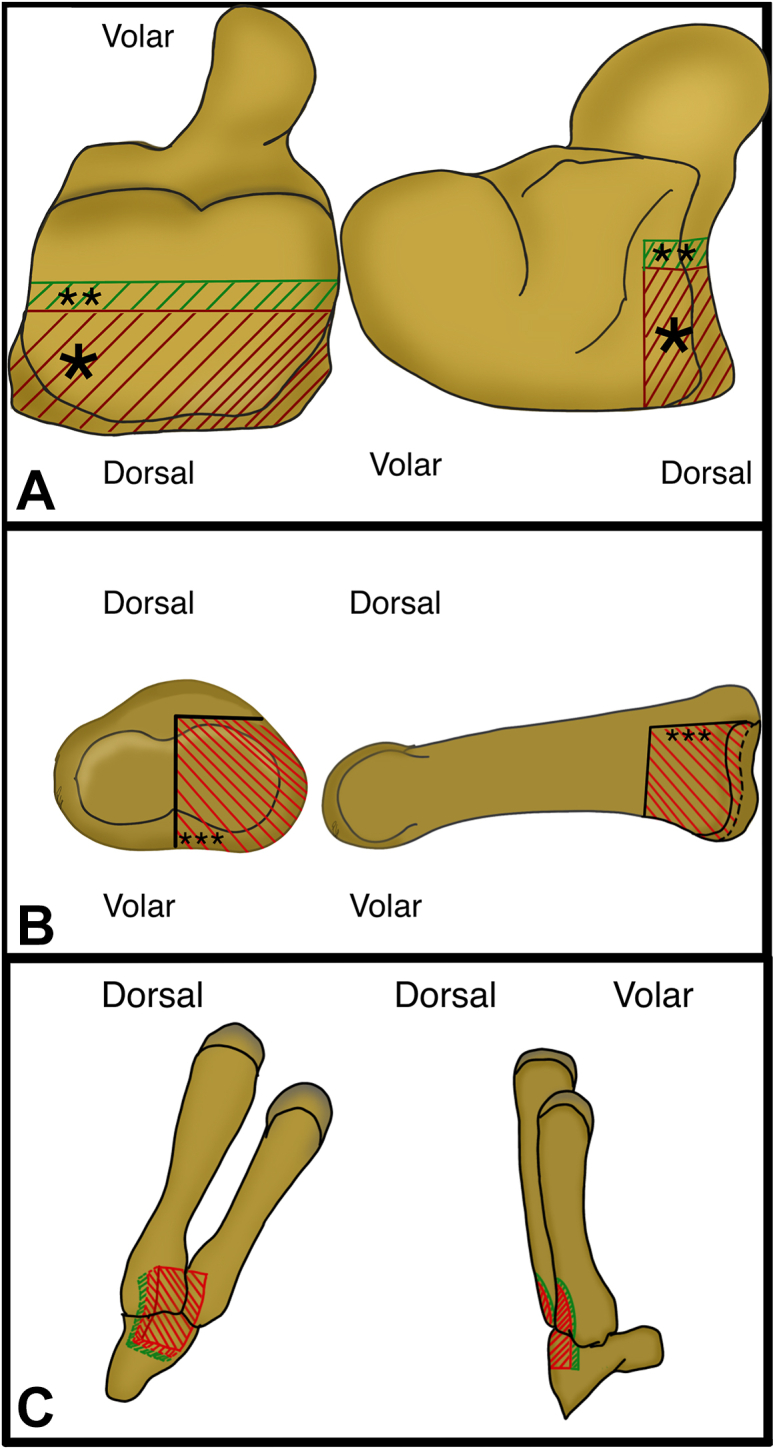
Schematic representation of the technique modification. **A** The picture shows the hamate ∗classical technique harvest ∗∗additional surface harvested. **B** ∗∗∗PIPJ defect. **C** The graft is harvested wider, and an only few millimeters of metacarpal base are excised.

Under regional anesthesia, a “shotgun approach” was performed to access the PIPJ (Fig. 2A). Articular joint defects were confirmed, and major chondropathy of P1 was excluded. To allow optimal graft harvesting, a small portion of the dorsal aspect of the base of the fourth and fifth metacarpal base was excised (Fig. 2B). To allow reconstruction of the entire ulnar articular surface of the P2 base, a modified version of the traditional hemi-hamate graft technique was performed, which included harvesting an anteroposterior graft that was approximately 3 mm in length and a portion of the hamate that was less wide (transversal plane) (Fig. 2C). The primary defect at the P2 base was debrided and regularized with a mini osteotome (Fig. 3A), and the graft was contoured to match the bone defect (Fig. 3B). Intraoperative stability was checked by manual maneuvering, and intraoperative fluoroscopy confirmed optimal graft fit, showing complete correction of the axial deviation and full restoration of passive joint flexion and extension and no impingement or articular slope. Fixation was carried out with a single 1.2 mm screw (Aptus Hand) (Fig. 4A,B); intraoperative fluoroscopy showed good reduction and stable fragment fixation. The volar plate was repaired with a 4–0 absorbable suture, and collateral ligament reconstruction was not necessary because of the dorsal proper collateral ligament integrity. The A3 pulley was sutured above the volar plate to reinforce its “buttress” effect (Fig. 4C). After the skin incision was closed, a dorsal splint was applied with the PIP joint flexed at approximately 50° to avoid joint hyperextension. A schematic of the modified technique is shown in Figure 5.

**Figure 6:**
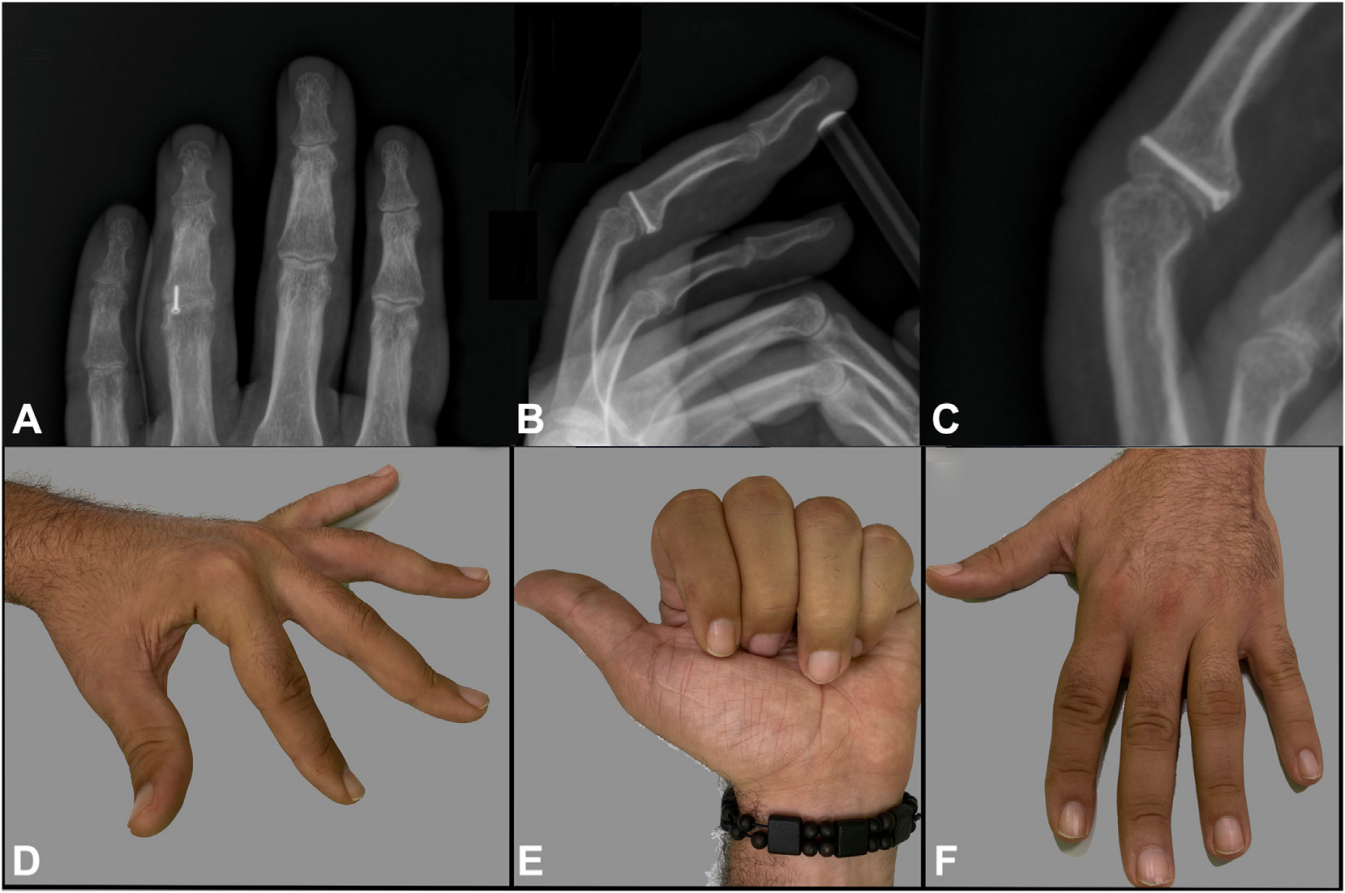
Clinical and radiographing assessment at the 3-month follow-up. **A** AP view. **B** LL view. **C** Detail on articular match and good osteointegration. **D** Lateral view at the 3-month follow-up. **E** Palmar view at the 3-month follow-up, corrected overlap. **F** Dorsal view at the 3-month follow-up, corrected fourth finger angulation.

Active range of motion exercises were started on the fourth postoperative day and were led by an experienced hand therapist. Eight weeks later, the patient underwent clinical and radiographic assessment (Fig. 6); imaging showed optimal graft osteointegration with consolidation at the bone-to-bone intersection.

**Figure 7:**
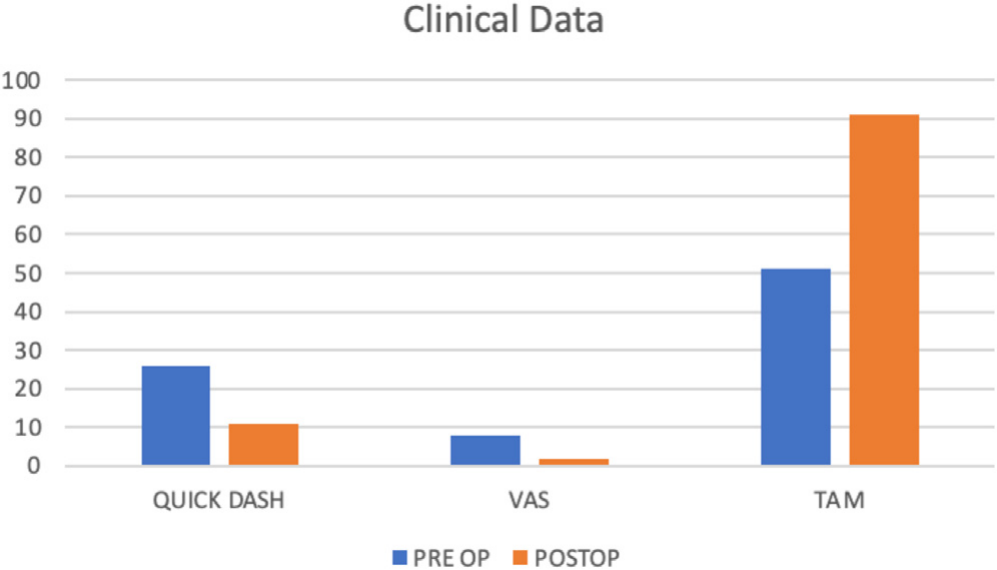
*Quick*DASH, VAS, and total active motion improvement at the 3-month follow-up.

We followed up with the patient clinically and radiologically after 3 months, and we collected data before surgery and 3 months after surgery. The data that were collected included the visual analog scale (VAS) score, *Quick*DASH score, and total active motion measurement. The data showed an overall improvement in all the parameters, with the VAS score improving from 8 to 2, *Quick*DASH from 26 to 11, and total active motion from 51° to 90° (Fig. 7). At the 3-month follow-up, the patient had returned to normal work. No discomfort at the donor site was reported.

Written informed consent was obtained from the patient for publication of this case report and accompanying images.

## Discussion

We note that PIP joint reconstruction represents a major challenge for surgeons, especially in patients with articular impaction and/or bone loss. Owing to the complexity and low occurrence of this injury pattern, no consensus exists on the best treatment; nevertheless, different strategies have been proposed in the literature. The hemi-hamate autograft was first described by Hastings and Carroll^2^ and has been proven to be a valuable option for patients with bone loss exclusively limited to the volar portion of the base of the second phalanx.^8^ Donor site morbidities are quite uncommon, consisting mainly of scar entrapment of the dorsalis branch of the ulnar nerve and carpometacarpal joint instability.^8^ Common complications include osteochondral graft fracture during harvesting or fixation and bony flexion blocking. A biomechanical study on cadaveric specimens by Elliot et al^9^ showed that the graft size should not be more than 3 mm larger than the native PIPJ articular surface to avoid flexion block.

Osteochondral autografts can be harvested from different donor sites, such as non-weight-bearing portions of the lateral knee condyle,^7^ a toe,^6^ or the costal arch. Despite the clinical success of these procedures, the most prominent drawback is the use of a second limb as the donor site.

Vascularized toe joint transfers are indicated for cases of highly compromised surrounding soft tissue, vascular impairment, or skeletal immaturity. Nevertheless, these procedures are time-consuming, have a high rate of reintervention, cause severe donor site morbidity, and require a high level of skill, and extensive training and experience. Furthermore, the patient needs to be suitable for this kind of microsurgical operation. A hemi-hamate autograft has been shown to be a valuable option for a volar lip PIPJ fracture, in cases of acute and chronic PIPJ fracture dislocation, offering good results in volar lip reconstruction with an average PIPJ flexion recovery of 77°.^10^ Little has been published about hemiarticular surface PIPJ reconstruction.

In this paper, we present a novel, nonvascularized osteochondral graft that is suitable for the reconstruction of bone loss involving the whole anteroposterior hemiarticular surface of the base of the P2. Compared with preoperative motion, a gain of 40% total active motion was achieved after surgery in addition to finger overlap correction and pain relief, with only a residual loss of 20° flexion in the distal phalangeal joint. The patient was satisfied overall and returned to his work 3 months after surgery. We can state that the hemi-*hemi*-hamate technique is a valuable option for the treatment of the whole hemi-ulnar or hemi-radial side of the articular surface of the base of P2. The technique has the advantages of the traditional hemi-hamate graft, using half of the usual hamate dorsal bone surface, with excellent outcomes, and avoids the need for secondary limb donor site and donor site impairment.
